# Alemtuzumab induction in pediatric kidney transplantation

**DOI:** 10.1111/petr.12048

**Published:** 2013-02-26

**Authors:** Michael M Kaabak, Nadezda N Babenko, Dmitry V Samsonov, Valery A Sandrikov, Alexey A Maschan, Alan K Zokoev

**Affiliations:** 1 Organ Transplant Division, Russian Scientific Center of SurgeryMoscow, Russia; 2 Kidney Transplant Department, Russian Scientific Center of SurgeryMoscow, Russia; 3 New York Medical CollegeValhalla, NY, USA; 4 Diagnostic Division, Russian Scientific Center for SurgeryMoscow, Russia; 5 Federal Clinical Research Center for Pediatric Hematology, Oncology and ImmunologyMoscow, Russia

**Keywords:** alemtuzumab, pediatric kidney transplantation, induction therapy, steroid free immunosupression

## Abstract

Recipient parenchymal lymphatic cells are crucial for direct and indirect pathways of allorecognition. We proposed that alemtuzumab, being infused several weeks pretransplant could eradicate peripheral lymphatic cells and promote donor-specific tolerance. We present here a single center, retrospective review of 101 consecutive living-donor kidney transplantations to pediatric patients aged from seven month to 18 yr, performed between September 2006 and April 2010. Immunosupression protocol included two 30 mg doses of alemtuzumab: first given 12–29 d prior to transplantation and second at the time of transplantation. Maintenance immunosupression was based on combination of low dose and wide range CNI and mycophenolate. Patients were followed for 3.8 ± 1.4 yr and protocol biopsies were taken one month, one, and three yr post transplant. The Kaplan–Meier graft and patient survival was 96% and 97% for one yr, 89% and 93% for three yr. Biopsy proven acute rejection developed in 26% patients at one yr and in 35% at two yr, no rejections occurred beyond two yr. We conclude that alemtuzumab pretreatment prior to living related donor kidney transplantation allows to reach satisfactory middle-term results in pediatric patients with wide range and low CNI concentrations.

Alemtuzumab (Campath-1H, MabCampath) is a humanized IgG1 monoclonal antibody directed against CD52, a glycoprotein expressed on mononuclear cells, including T and B lymphocytes, monocytes, and natural killer cells 1, 2. Alemtuzumab is the most powerful of the currently used lymphocyte-depleting agents; it brings about a rapid and sustained depletion of circulating and peripheral lymphocytes 3, 4. Maximal depletion of peripheral lymphocytes takes between two and 10 d and has been confirmed in both nonhuman primates and transplant patients 3, 5.

Alemtuzumab has been used as an induction agent in renal transplantation since the first (1998) report by Calne et al. 6, who demonstrated that the use of alemtuzumab induction allowed transplant recipients to be maintained on a low-dose cyclosporine monotherapy. Subsequent five-yr follow-up confirmed that under this immunosuppressive protocol, the patient and graft survival was similar to that achieved with conventional therapy 7. A few years later, the Pittsburgh group demonstrated promising three-yr survival rates with low-dose tacrolimus monotherapy after alemtuzumab induction 8. According to UNOS, alemtuzumab induction was utilized in 14.1% of all kidney transplantations performed in the United States between 2000 and 2010 (based on OPTN data as of January 14, 2011). In a recently published prospective randomized trial 9, alemtuzumab was associated with lower rates of acute rejection than basiliximab in low immunological risk patients and was associated with similar efficacy as compared with rabbit anti-thymocyte globulin in high-risk patients. The superiority of alemtuzumab over daclizumab was also demonstrated in a randomized trial 10. Calne and Watson's review 11 suggested that alemtuzumab induction reduced the dosage required for maintenance immunosuppression; there was an increased proportion of regulatory T cells after alemtuzumab use.

The use of alemtuzumab in pediatric kidney transplantation is relatively limited. The first report of four patients was unfavorable: rejection was seen in three of four patients, including two antibody-mediated rejections 12. The largest series of pediatric patients was published by the Pittsburgh group, whose protocol included pretreatment of recipients with a single dose of alemtuzumab as well as tacrolimus monotherapy 13. An average four-yr follow-up of 42 pediatric patients showed promising results in terms of safety, efficacy, and tolerability 14.

We modified the protocol utilized by the Pittsburgh group. Our patients received two doses of alemtuzumab, pretreatment with alemtuzumab two to three wk before the transplantation and the second alemtuzumab dose on the day of transplantation. The rationale for this specific protocol is an attempt to achieve maximal peripheral lymphocyte depletion during and after the transplantation. The depletion of recipient and donor antigen-presenting cells is expected to induce the abrogation of direct and indirect allorecognition and to impair costimulatory signaling 15, 16.

This study evaluates the advantages and disadvantages of this strategy with an emphasis on the analysis of recipient survival, graft loss, acute rejection, and infections.

## Materials and methods

This single-center, retrospective review covered alemtuzumab induction therapy for 101 consecutive living donor kidney transplantations in pediatric patients between seven months and 18 yr of age, performed between September 2006 and April 2010 at the Russian Scientific Center of Surgery, Moscow, Russia. The alemtuzumab induction protocol was reviewed and approved by our institution's Ethics Committee, and informed consent was received from the patients' parents or guardians. Our institution used a two-dose alemtuzumab induction regimen: one dose of 30 mg 12–29 d prior to the transplantation (18.0 ± 3.1) and one dose at the time of transplantation, 30 mg for children over 10 kg and 15 mg for children weighing 10 kg or less. The patients were followed for 3.8 ± 1.4 yr. Two patients were unavailable for follow-up because of a change of residence 213 and 912 d post-transplant.

**Fig. 1:**
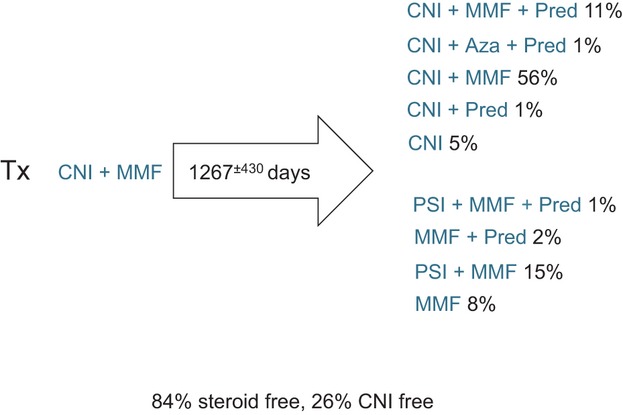
Evolution of maintenance immunosuppression.

All patients were initially treated with a combination of a CNI, MMF, and steroids. The choice of CNI (cyclosporine or tacrolimus) was initially dependent on drug availability; beginning in April 2009, we began a randomized trial of cyclosporine or tacrolimus (clinicaltrials.gov identifier: NCT01346397). The trough levels of tacrolimus were adjusted to 8–12 ng/mL for the first 10 d and 2–8 ng/mL starting from day 11. The levels of cyclosporine were measured before dose intake, at one and three h after administration of the dose and adjusted for the target AUC: 3500–4500 ng/mL/h and C0 100–200 ng/mL until day 10; AUC around 2500 and C0 75–150 until day 30; AUC 1500–2000 and C0 50–100 thereafter. AUC was calculated using a Gaspary equation 17. All patients received 10 mg/kg of methylprednisolone intravenously before reperfusion. From day one, all patients received 60 mg/m^2^ of prednisone (maximal dose 80 mg). Steroids were discontinued in patients with good early graft function when the desired level of CNI was reached, usually by day five after transplantation. In patients with impaired graft function, steroids were withdrawn if weekly graft biopsies did not show any signs of rejection. MMF was introduced at a dose of 1200 mg/m^2^/d after the resolution of neutropenia caused by the induction therapy (white blood cell count above 3.0 × 10^6^/mL). MMF was discontinued and replaced by azathioprine in one patient because of intolerable side effects (diarrhea). In cases when reduction in immunosuppression was clinically indicated because of acute CMV or BK infection or rising EBV-PCR titers, the first choice was the initial reduction in the CNI dosage by 50%. If further reduction was necessary and proteinuria did not exceed 1 g/d, the CNI was replaced with a PSI. Both everolimus and sirolimus were used; the choice was dependent on drug availability, with the target trough blood level at 2–8 ng/mL for both drugs. In case of proteinuria >1 g/d, monotherapy with MMF was also allowed (Fig. 1).

ABOi transplant recipients received a similar induction and maintenance protocol except for pretransplant plasmapheresis, which was performed in six of eight ABOi children to achieve the target levels of isoagglutinins. The number of plasmapheresis treatments was low: 1.5 ± 1.1 per patient (Table 1); the volume of removed plasma during one session was as high as 150% of the total volume of circulating plasma. After the last plasmapheresis, the titer of isoagglutinins was checked twice, on two consecutive days. If the titer was low (below eight, according to the tube technique method) and was not rising, we performed transplantation. Two children had a low original isoagglutinin titer and did not need plasmapheresis.

**Table 1:** Donors' and recipients' demography

	n	(%)		
Donor	101	100.0		
Female/male	74/27	73.3/26.7		
Mean donor age	39 ± 8.1			

			HLA mismatch	
			
Donor relationship torecipient			I class	II class
All	101	100.0	1.6 ± 0.7	0.9 ± 0.6
Parent	83	82.2	1.5 ± 0.6	0.8 ± 0.5
Sibling	1	1.0	0	0
Other related	17	16.8	1.9 ± 1.0	1.5 ± 0.6
Genetically unrelated	0	0.0		
Recipient	101	100		
Diagnosis				
Congenital dysplasia	23	22.8		
Reflux nephropathy	29	28.7		
FSGS	6	5.9		
Obstructive (valves + hydronephrosis)	5	5.0		
Congenital nephrotic syndrome	4	4.0		
HUS	9	8.9		
Alport syndrome	3	3.0		
Cystinosis	1	1.0		
Other diagnosis (vasculitis, TIN, cortical necrosis, Fanconi, glomerulonephritis)	14	13.9		
Unknown	7	6.9		
Female/male	52/49			
Mean recipient age	10.7 ± 5.1			
PRA > 30	5	5.0		
Primary graft	100	99.0		
Secondary graft	1	1.0		
ABOi, patients no.	8	7.9		
Plasma exchange pre-Tx, patients no.	6	75.0		
Plasma exchange pre-Tx, procedures per patient no.	1.5 ± 1.1			
Isoagglutinin titer				
Original	5.8 ± 2.3			
Pre-Tx	2.3 ± 1.5			

PRA, panel reactive antibodies.

Protocol biopsies were performed on day 30, one, and three yr after transplantation. Graft biopsies were performed also to clarify the reason for kidney dysfunction, which was defined as the elevation of either serum creatinine or proteinuria over 30% above baseline, on two consecutive tests. Renal allograft rejection was diagnosed by allograft biopsy. Rejection episodes were treated by a switch from CsA to Tacro and then by a patient-specific combination of either three doses of high-dose IV steroids every 48 h (500 mg/m^2^ during first month and 400 mg/m^2^ thereafter) or three doses of IV steroids followed by the addition of an oral steroid recycle starting with 25 mg/m^2^ of prednisone with a rapid taper or oral steroid re-administration. Alternatively, an oral steroid recycle was used without IV steroids, with the addition or increase in the MMF dosage. The severity and acuteness of the rejection, anticipated side effects of medications, and available diagnostic and treatment options determined the design of a specific antirejection treatment for each individual patient.

Viral infections, CMV, BKV, and EBV were defined as the presence of viremia (for BKV, also viruria) by DNA capture or more recently by quantitative PCR analysis.

All patients received prophylaxis against PCP with daily TMP/sulfa for six months. Viral prophylaxis with valganciclovir was given to all patients regardless of their pretransplant CMV and EBV status for three wk after transplantation, with subsequent CMV-PCR monitoring every month for the first year and every three months thereafter. In the event of detectable CMV viremia, preemptive therapy with valganciclovir was re-introduced.

Patient and graft survival, the incidence of rejection, and the incidence of viral infections were analyzed for the whole group as well as for the cyclosporine and tacrolimus groups.

## Results

### Patient characteristics and survival

The recipient and donor demography is summarized in Table 1, along with the HLA mismatches in each category. The major causes of ESRD were reflux nephropathy in 28.7% and congenital dysplasia in 22.8%. Diseases that can recur in the transplant were found in 5.9% with FSGS, 8.9% with HUS. The average HLA mismatch was 2.5 ± 1.0.

Eight recipients received an ABOi kidney, A to 0 (n = 3) and B to 0 (n = 3), A to B (n = 1) and B to A (n = 1); their characteristics are summarized in Table 1.

The Kaplan–Meier patient actuarial survival at one, two, and three yr was 97.0 ± 2.0%, 94.0 ± 3.5%, and 93.0 ± 2.1%. There were eight (7.9%) recipient deaths with a functioning graft (Table 2 Part a).

**Table 2:** Mortality and particular postoperative courses

No.	Gender	Age at tx	Postoperative day	Cause of death
(a) Causes of death in 8 of 101 alemtuzumab-treated patients				
1	Girl	7 yr	452	Respiratory failure due to ascending paralysis
2	Boy	7 months	165	Brain edema due to electrolyte disorders caused by acute kidney obstruction with subsequent urine outflow restoration
3	Girl	8 yr	1457	Meningococcal meningoencephalitis
4	Girl	3 yr	420	Unknown
5	Boy	2 yr	548	PCP
6	Girl	11 months	26	Pneumonitis, pathogen not specified
7	Girl	7 yr	891	Pneumonitis, pathogen not specified
8	Boy	15 yr	273	Pneumonitis, pathogen not specified

(b) Clinically apparent EBV and CMV infections and PCP				Outcome
1	Boy	4 yr	EBV-associated flu like syndrome 6 months post-transplant, resolved after switch from Tacro + MMF to Rapamune	4 yr, excellent graft function
2	Boy	2 yr	PCP developed 1 month post-transplant, completely resolved on trimethoprim-sulfametoxasole	3 yr, impaired graft function (GFR 20 mL/min)
3	Boy	6 yr	CMV-associated pneumonia on day 230, 4 wk apart of steroid-resistant rejection treatment with Campath, completely resolved with 4-wk valganciclovir	3 yr, excellent graft function
4	Boy	4 yr	CMV-associated enteritis 1 yr post-transplant, resolved with 2-wk valganciclovir	3 yr, excellent graft function

(c) Reasons for not withdrawal the steroids in 5 of 101 alemtuzumab-treated patients				
1	Boy	7 months	Gastrointestinal infection on day 7 with acute graft failure, CNI was suspended for 4 days, graft function recovered promptly, CNI target level was achieved slowly and proper time for steroid withdrawal was missed				
2	Girl	8 yr	Delayed graft function until day 28, we preferred to left her on steroids then undergo every week biopsy				
3	Boy	5 yr	Decortication due to profound and prolonged hypoglycemia, PSI was used instead CNI to avoid neurotoxicity				
4	Girl	10 yr	Slow graft function (normal creatinine on day 21) caused by artery thrombosis with restoration of blood flow within 1 h	Vulnerability to rejection due to ischemic injury made us continue steroids in these two children
5	Girl	13 yr	Slow graft function (normal creatinine on day 44) caused by venous kinking with restoration of blood outflow within 4 h	

### Graft survival and function

The graft survival at one, two, and three yr was 96.0 ± 2.0%, 92.1 ± 4.0%, and 89.1 ± 3.6%. The mean serum creatinine (mg/dL) was 1.0 ± 0.5, 1.1 ± 0.7, 1.2 ± 0.8, and the GFR (measured by the classic Schwartz formula) was 91.6 ± 33.7, 84.0 ± 37.2, and 82.8 ± 30.5 mL/min at one, two, and three yr, respectively. Proteinuria (mg/24 h) was 271 ± 315, 526 ± 586, and 180 ± 301 at one, two, and three yr. Immediate function was observed in 93% of allografts. Six children required one session of hemodialysis during the first postoperative week, and one child received dialysis for four wk until graft function recovered. The loss of five renal allografts was related to graft artery thrombosis in one patient on day 0, noncompliance in three adolescent patients on days 433, 836, and 1018 post-transplant and to a refractory rejection in one patient on day 1409.

Mean follow-up of the ABOi recipients over 3.3 ± 0.6 yr showed no graft loss or patient death. One child has significantly impaired graft function because of a refractory rejection, with a serum creatinine of 6.4 mg% and a GFR of 11 mL/min at 2.5 yr post-transplant. She is being reevaluated for retransplantation. The other seven ABOi recipients have good renal function. The mean serum creatinine (mg/dL) at last follow-up was 1.1 ± 0.3, with a GFR of 70.7 ± 21.6 mL/min, and no significant proteinuria (130 ± 112 mg/24 h).

### Immunosuppressive therapy

All patients received intravenously of methylprednisolone 10 mg/kg before reperfusion. From day one, all patients received 60 mg/m^2^ of prednisone (maximal dose was 80 mg). Steroids were discontinued after 7.0 ± 6.4 d in 96 of 101 patients with good primary graft function after achieving the target levels of the CNI. Reasons for steroid continuation are indicated in Table 2 Part b.

CNIs (cyclosporine in 63 patients and tacrolimus in 36 patients) were started on day zero (0.0 ± 0.3). Two patients did not receive a CNI (one patient was started on sirolimus because of severe central nervous system dysfunction and was switched to cyclosporine at day 50; another patient received an HLA identical kidney from his brother).

In the cyclosporine group (63 patients at day one), 19 (30%) patients were subsequently switched to tacrolimus 375 ± 281 d post-transplant because of rejection. Three yr post-transplantation, 15 (24%) and 48 (76%) children were receiving CNI–free and steroid-free immunosuppression, respectively. In the tacrolimus group three yr post-transplantation, eight (22%) and 33 (92%) children were receiving CNI–free and steroid-free immunosuppression, respectively. Indications for discontinuation of the CNI were clinical evidence of overimmunosuppression, including recurrent viral infections, either clinical or laboratory: positive NATs for EBV, CMV, BKV in blood, high levels (>10^7^) of BKV in urine, and progressive morphological signs of nephrotoxicity.

### Rejection

**Fig. 2:**
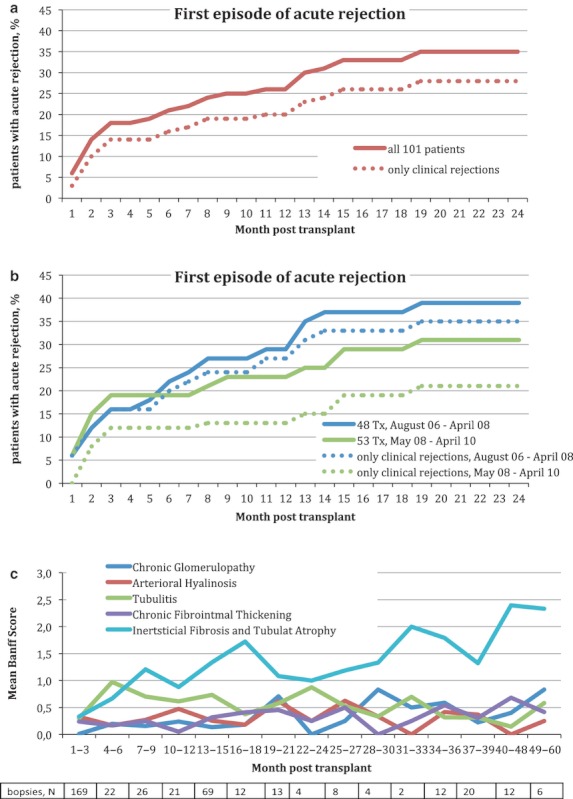
(a) Acute rejection in 101 alemtuzumab treated patients; (b) patients stratified by time of transplantation. Incidence of clinical rejection depicted by dotted lines. (c) Mean Banff scores for chronic interstitial fibrosis, tubular atrophy, arteriolar hyalinosis, chronic fibrointimal thickening, and chronic glomerulopathy.

The incidence of acute rejection is shown in Fig. 2. Any tubulitis, including subclinical changes, was diagnosed as acute rejection (subclinical seven of 37% or 19% of all rejections). The incidence of cumulative biopsy-proven rejection at one, two, and three yr was 26%, 35%, and 35%, respectively. The degree of rejections according to Banff 97 score was as follows: among 37 rejections, 14 (38%) were borderline; 19 (51%), 1a; 1 (3%), 1b; and 3 (8%), 2a. Among 49 patients operated between August 2006 and April 2008, acute rejection developed in 29% and 39% for one and two yr, respectively, while among 52 patients operated between May 2008 and April 2010, the figure was 23% and 31%, respectively (Fig. 2b). We attributed the decreasing incidence to the accumulation of experience with alemtuzumab and more timely MMF administration; fear of overimmunosuppression during the first few months may also have played a role.

Treatment of acute rejection: 25 of 37 acute rejection episodes were treated with steroids (intravenously and/or orally). The remaining 12 patients were treated only with increased oral immunosuppression (switching from cyclosporine to tacrolimus or increasing the MMF dose). Two patients had steroid-resistant rejections and were successfully treated with alemtuzumab on days 206 and 383. In four patients, rejections were irreversible (three noncompliant patients) and led to graft loss on days 433, 836, 1018, and 1409.

Mean Banff scores for IFTA, arteriolar hyalinosis, chronic fibrointimal thickening, and chronic glomerulopathy are presented in Fig. 2c. The number of long-term biopsies was not sufficient to provide a definite conclusion, but we can see a trend toward a slight increase in IFTA and a very moderate presence of other chronic changes and tubulitis over time post-transplant.

### Infections

**Fig. 3:**
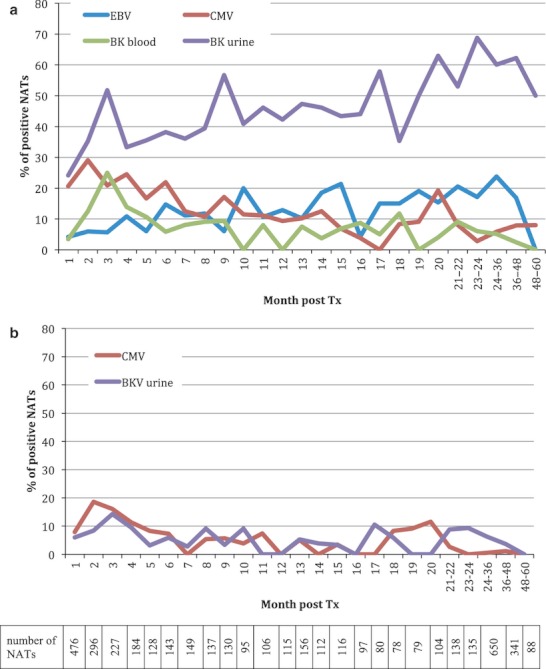
(a) Positive nucleic acid tests regardless of viral bodies concentrations. (b) BK viruria more than 10^7^ copies/mL and CMV of 10^3^ or more copies/mL – generally accepted as “dangerous” viral loads – considered as positve.

All patients received prophylaxis with valganciclovir for three wk after transplantation. CMV, EBV, and BK viremia were monitored monthly during the first post-transplant year and every three months thereafter. In cases of detectable CMV viremia, preemptive therapy with valganciclovir was re-introduced. CMV and BKV viremia were highest during the first three months and reached 30% and 25% of positive NATs. Beyond one yr, the level of CMV did not exceed 10%, and BKV viremia was <5%. EBV viremia occurred with increasing frequency and reached 20% by year two. The incidence of BK viruria also increased with time, exceeding 50% by year two (Fig. 3 Part 1) and correlated well with the previously observed prevalence of BKV infection in our population 18. We did not detect any cases of PTLD; however, two children developed lymphadenopathy associated with EBV viremia that improved after discontinuation of the CNI. The incidence of EBV- and CMV-associated infections, as well as PCP, is summarized in Table 2 Part c. Immunosuppression was decreased in cases of BK viruria of above 10^7^ copies/mL or repeated BK or EBV viremia as well as recurrent CMV viremia.

**Fig. 4:**
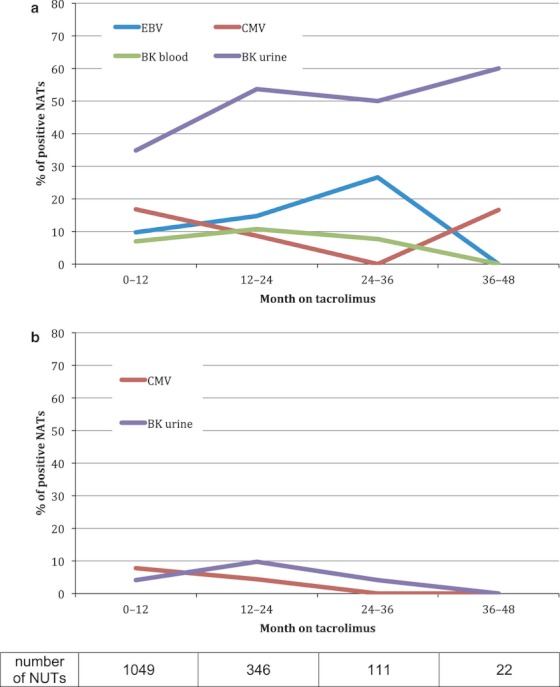
(a) Tacrolimus treated patients at the time of sampling. Positive nucleic acid tests regardless of viral load, as a function of time on tacrolimus. (b) Tacrolimus treated patients at the time of sampling. BK viruria more than 10^7^ copies/mL and CMV of 10^3^ or more copies/mL – generally accepted as “dangerous” viral loads – considered as positve.

**Fig. 5:**
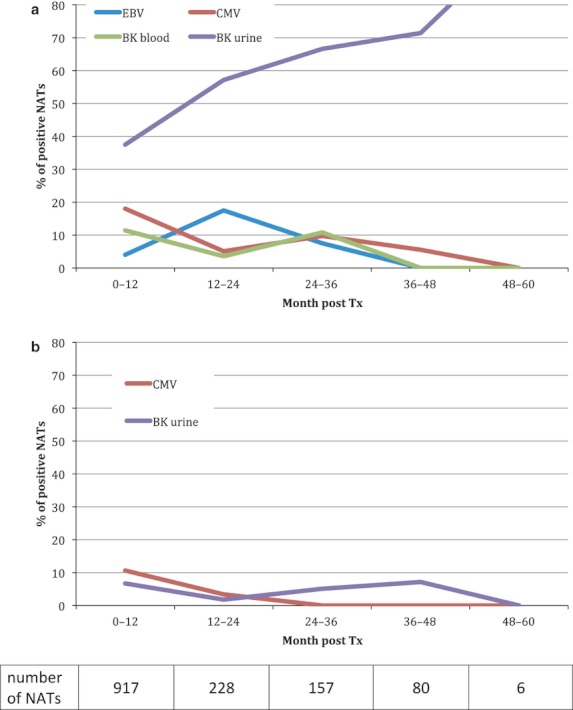
(a) Cyclosporine treated patients at the time of sampling. Positive nucleic acid tests regardless of viral load, as a function of time on cyclosporine. (b) Cyclosporine treated patients at the time of sampling. BK viruria more than 10^7^ copies/mL and CMV of 10^3^ or more copies/mL – generally accepted as “dangerous” viral loads – considered as positve.

**Fig. 6:**
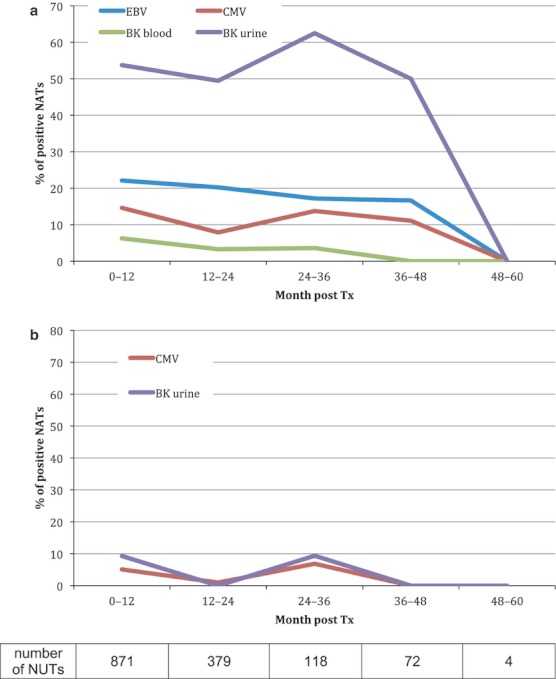
(a) CNI-free patients at the time of sampling. Positive nucleic acid tests regardless of viral load, as a function of time without CNI. (b) CNI-free patients at the time of sampling. BK viruria more than 10^7^ copies/mL and CMV of 10^3^ or more copies/mL – generally accepted as “dangerous” viral loads – considered as positve.

Figs. 4–6 demonstrate the evolution of viral loads depending on immunosuppression. It is evident that both tacrolimus- and cyclosporine-based immunosuppression were associated with an increased BKV infection rate, while CNI-free immunosuppression was associated with stabilization and a declining trend. The high viral infection rate at the beginning of CNI-free period is explained by a previous CNI exposure. There were no definite cases of allograft nephropathy associated with BKV.

## Discussion

Antibody induction therapy is commonly utilized in renal transplant patients. In comparison with other induction therapies, alemtuzumab has been shown to be more effective in preventing acute rejection episodes 9 and allows lower doses of CNI monotherapy 6, 8. In our study, we implemented the Pittsburgh group strategy, which suggests that lymphoid depletion prior to graft revascularization might result in the development of partial tolerance to the allograft. We enhanced this strategy by giving the first dose of alemtuzumab two to three wk prior to living donor kidney transplantation and the second dose on the day of transplantation. The resulting peripheral lymphocyte depletion should prevent the activation of alloimmune response via direct and indirect allorecognition. Because IL-2 inhibition may interfere with tolerance induction, our second goal was to minimize CNI exposure in the post-transplant period. This theoretically justified strategy is also strengthened by the practical needs of the majority of Russian kidney recipients. Most of our patients live far away from the transplant center, and the availability of local drug level monitoring is often limited. The disadvantage of this strategy is the early administration of immunosuppression, with the need to perform major surgery in immunosuppressed children. In addition, the optimal use of mycophenolate is compromised by the alemtuzumab-induced neutropenia. Mycophenolate dosage has to be increased proportionally as the bone marrow recovers, and this required prolonged and careful patient monitoring. We attribute the trend of decreased rejection rates (Fig. 2b) in more recent cases to the accumulation of experience and proper mycophenolate administration.

This study represents the largest single-center series of pediatric kidney transplant recipients receiving alemtuzumab pretreatment. The three-yr patient and graft survival rates were 93.0 ± 2.1% and 89.1 ± 3.6%, respectively. An historical control group of 37 live donor pediatric kidney transplantations performed at our center between November 2002 and September 2006 with Zenapax induction demonstrated a trend toward lower three-yr patient and graft survival rates: 88.9 ± 6.1% and 81.1 ± 6.3%, correspondingly. The graft survival rate in alemtuzumab group is comparable to and patient survival is lower than living donor kidney transplant recipients reported by NAPRTCS 19, where three-yr patient and graft survival of live donor pediatric kidney transplant recipients was 97.9 ± 0.5% and 91.3 ± 0.6%, respectively, for transplants performed between 1996 and 2010. Lower patient survival and comparable graft survival can be attributed to inadequate availability of dialysis to children in some Russian regions; in those cases, the loss of graft function sometimes means patient death. The high late mortality rate to infections (Table 2 Part a) can be partially explained by the absence of vaccinations in our patients. Despite ESRD, intensive vaccination is not an established rule in our country. We believe that pretransplant vaccination against meningococcal, pneumococcal, and hemophilus infections could have saved lives in patients 3, 6, 7, and 8. Aggressive pre- and post-transplant vaccinations have been widely used since 2010, and we expect a positive change in patient survival rates. Our protocol allowed us to achieve non-death-censored graft survival rates comparable to those observed by NAPRTCS, despite infection-induced mortality.

The majority of patients received MMF and a CNI, with early discontinuation of steroids. The incidence of biopsy-proven rejection (including borderline and subclinical) at one, two, and three yr was 26%, 35%, and 35%, respectively. The incidence of clinical Banff 1a or worse rejection at one, two, and three yr was twice less – 13%, 18%, and 18%. Rejection was less frequent in tacrolimus-treated patients; overall, three-yr incidence was 19% for all rejections (including borderline and subclinical) and 10% for clinical Banff 1a or worse rejections. We would like to be careful with conclusions when comparing two CNIs in this article, because majority of patients are not randomized and duration of follow-up in tacrolimus group is shorter.

The term “rejection” may have a different meaning in different studies. Thus, the most cited publication on kidney transplantation morphology, describing long-term follow-up of 120 patients, reported rejection rate of 71.4%. However, these rejections had only a limited effect on grafts, because the 10-yr death-censored kidney graft survival rate was 95.2% 20. This controversy, as well as a high discrepancy in reported rejection rates, can be explained by different definitions of rejection. In our patients, the criteria for rejection were any morphological signs according to Banff classification 21, 22, regardless of graft function.

There were six patients with FSGS and nine patients with HUS in our cohort. Among the FSGS patients, no recurrence was seen; two adolescent girls lost a graft to noncompliance: one developed acute rejection eight months post-transplant, refused steroid treatment, and lost the graft seven months later; the second suffered depression 14 months post-transplant after the death of her grandmother, refused oral intake of food, water, and medications, and lost the graft due to rejection. In the HUS patients, one three-yr-old boy had an early recurrence with acute renal failure that was partially resolved after six months, and subsequent graft function was maintained for three yr with a GFR of around 30 mL/min. He then developed a left pulmonary artery thrombosis. The course of ensuing thrombolysis was complicated with graft rupture and abdominal bleeding, and the graft was removed. The remaining eight HUS patients have had a favorable postoperative course. The details of long-term follow-up in FSGS and HUS patients are summarized in Table 3.

**Table 3:** Post-transplant course in FSGS and HUS patients

	FSGS	HUS
Number of patients	6	9
Patients with rejections	2	1
Long-term graft survival	4 of 6	8 of 9
At last control, days post-transplant	1390 ± 569	1080 ± 146
Graft function		
Serum creatinine, mg%	0.7 ± 0.2	0.8 ± 0.2
Proteinuria, mg/24 h	45 ± 55	69 ± 48
Calculated GFR, Schwartz formula, mL/min	132 ± 42	101 ± 26
Immunosuppression		
PSI + MMF	2	3
Tacrolimus + MMF	1	3
Cyclosporine + MMF	1	1
Pred + CsA + Aza		1

The major concern of alemtuzumab induction in pediatric transplantation is safety. The incidence of serious infections was relatively low, with clinically apparent CMV and EBV infections in 2% and 1%, respectively (Table 2 Part b). However, the subclinical disease detected by rising titers of viral load was detected in higher levels (Fig. 3) and required a modification of immunosuppression. There were no cases of PTLD, possibly because alemtuzumab also affects B cells.

At three yr, 84% of patients remained steroid free and 26% of patients were CNI free (Fig. 1). Apart from lower viral load and less nephrotoxicity, CNI-free immunosuppression can result in decreased donor-specific recipient T-cell reactivity and can accelerate the development of recipients' FOXP3+ Tregs in renal transplant recipients, perhaps enhancing the potential for graft acceptance 23, 24.

Separate analysis of the outcomes in children who initially received cyclosporine or tacrolimus is summarized in Table 4, Figs. 4 and 5. However, conclusions should be drawn carefully when interpreting the results, because the majority of these patients are not randomized. Thus, three-yr graft survival rates are superior in the tacrolimus group, but the follow-up duration is shorter. The rejection rate was significantly lower in tacrolimus patients. The viral infection incidence is presented as percentage of positive NATs (Figs. 4–6). The EBV infection rate in tacrolimus-treated patients was 27% between years 2 and 3 (Fig. 4). New-onset diabetes mellitus was seen in two (5% and 6%) tacrolimus-treated patients during treatment for rejection with intravenous and oral steroids; in both cases, insulin was used for glucose control. After tapering of oral steroids, insulin dependence diminished, and after six wk in the former case and three months in the latter, the children maintained a normal glucose metabolism without antidiabetic medications.

**Table 4:** Results of kidney transplantation in children under alemtuzumab induction and received initially maintenance immunosuppression based either on cyclosporine or tacrolimus

	Cyclosporine	Tacrolimus
Number of patients	63	36
Acute rejections, patients (%)	27 (43)	7 (19)
Follow-up, d	1390 ± 506	1112 ± 305
Three-yr survival, %		
Patient	90 ± 4	97 ± 5
Graft	83 ± 7	97 ± 5

Obviously, this report has several important limitations. This is a retrospective analysis with no control groups. Prolonged follow-up will be required to assess long-term patient and graft survival and function, as well as the rate of infectious and malignant complications. Prospective trials evaluating alemtuzumab-pretreatment protocols with different post-transplant immunosuppressive regimens are warranted.

## Abbreviations

ABOi: ABO-incompatible
AUC: area under the curve
BKV: BK virus
CMV: cytomegalovirus
CNI: calcineurin inhibitor
CsA: cyclosporine A
EBV: Epstein–Barr virus
ESRD: end-stage renal disease
FSGS: focal segmental glomerulosclerosis
GFR: glomerular filtration rate
HLA: human leukocyte antigen
HUS: hemolytic uremic syndrome
IFTA: interstitial fibrosis and tubular atrophy
MMF: mycophenolate mofetil
NATs: nucleic acid test
PCP: pneumocystis carinii pneumonia
PCR: polymerase chain reaction
PSI: proliferative signal inhibitor
PTLD: post-transplant lymphoproliferative disorder
